# Urinary neutrophil gelatinase-associated lipocalin and plasma IL-6 in discontinuation of continuous venovenous hemodiafiltration for severe acute kidney injury: a multicenter prospective observational study

**DOI:** 10.1186/s13613-023-01137-6

**Published:** 2023-05-15

**Authors:** Yohei Komaru, Moe Oguchi, Tomohito Sadahiro, Taka-aki Nakada, Noriyuki Hattori, Takeshi Moriguchi, Junko Goto, Hidetoshi Shiga, Yoshihiko Kikuchi, Shigeo Negi, Takashi Shigematsu, Naohide Kuriyama, Tomoyuki Nakamura, Kent Doi

**Affiliations:** 1 Department of Hemodialysis and Apheresis, The University of Tokyo Hospital, Tokyo, Japan; 2 Department of Emergency and Critical Care Medicine, Tokyo Women’s Medical University Yachiyo Medical Center, Chiba, Japan; 3 Department of Emergency and Critical Care Medicine, Chiba University Graduate School of Medicine, Chiba, Japan; 4 Department of Emergency and Critical Care Medicine, University of Yamanashi, Faculty of Medicine, Yamanashi, Japan; 5 Emergency and Intensive Care Center, Teikyo University Chiba Medical Center, Chiba, Japan; 6 Department of Nephrology, Wakayama Medical University, Wakayama, Japan; 7 Department of Anesthesiology and Critical Care Medicine, Fujita Health University School of Medicine, Aichi, Japan; 8 Department of Emergency and Critical Care Medicine, The University of Tokyo Hospital, 7-3-1 Hongo, Bunkyo-ku, Tokyo, 1138655 Japan

**Keywords:** Acute kidney injury, Continuous venovenous hemodiafiltration, Discontinuation, Neutrophil gelatinase-associated lipocalin, Interleukin 6

## Abstract

**Background:**

Patients with severe acute kidney injury (AKI) who require continuous venovenous hemodiafiltration (CVVHDF) in intensive care unit (ICU) are at high mortality risk. Little is known about clinical biomarkers for risk prediction, optimal initiation, and optimal discontinuation of CVVHDF.

**Methods:**

This prospective observational study was conducted in seven university-affiliated ICUs. For urinary neutrophil gelatinase-associated lipocalin (NGAL) and plasma IL-6 measurements, samples were collected at initiation, 24 h, 48 h after, and CVVHDF discontinuation in adult patients with severe AKI. The outcomes were deaths during CVVHDF and CVVHDF dependence.

**Results:**

A total number of 133 patients were included. Twenty-eight patients died without CVVHDF discontinuation (CVVHDF nonsurvivors). Urinary NGAL and plasma IL-6 at the CVVHDF initiation were significantly higher in CVVHDF nonsurvivors than in survivors. Among 105 CVVHDF survivors, 70 patients were free from renal replacement therapy (RRT) or death in the next 7 days after discontinuation (success group), whereas 35 patients died or needed RRT again (failure group). Urinary NGAL at CVVHDF discontinuation was significantly lower in the success group (93.8 ng/ml vs. 999 ng/ml, *p* < 0.01), whereas no significant difference was observed in plasma IL-6 between the groups. Temporal elevations of urinary NGAL levels during the first 48 h since CVVHDF initiation were observed in CVVHDF nonsurvivors and those who failed in CVVHDF discontinuation.

**Conclusions:**

Urinary NGAL at CVVHDF initiation and discontinuation was associated with mortality and RRT dependence, respectively. The serial changes of urinary NGAL might also help predict the prognosis of patients with AKI on CVVHDF.

**Graphical Abstract:**

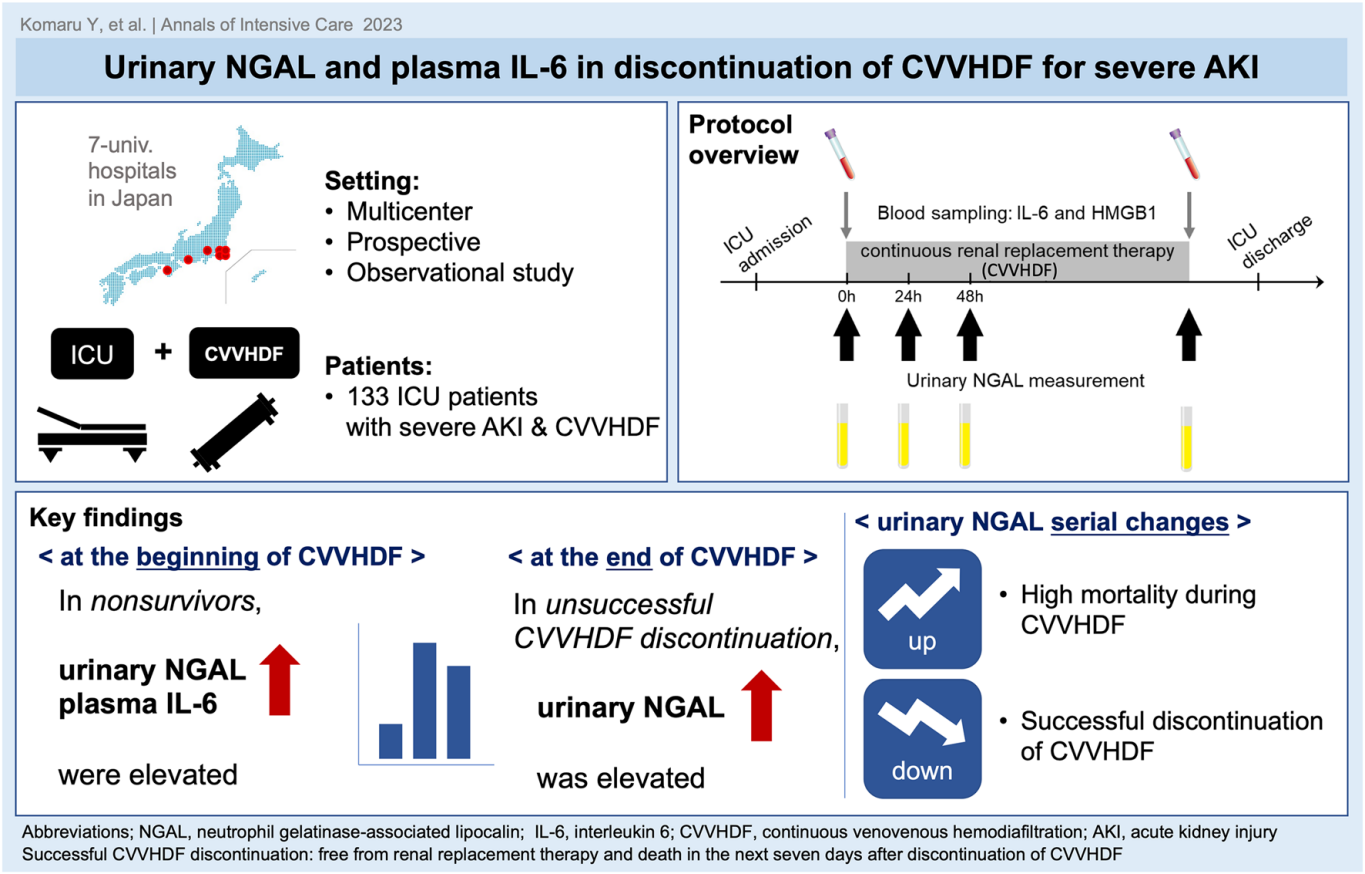

**Supplementary Information:**

The online version contains supplementary material available at 10.1186/s13613-023-01137-6.

## Background

Critically ill patients in intensive care unit (ICU) frequently develop acute kidney injury (AKI), which has a significant impact on their outcomes including mortality. The AKI incidence in ICU has reportedly reached 30–60% worldwide [1, 2]. Continuous venovenous hemodiafiltration (CVVHDF) is an effective treatment widely used for patients with severe AKI in ICU to correct uremic milieu, electrolyte abnormalities, and fluid imbalance. Even with the current advances in multidisciplinary care in ICU, patients with AKI requiring renal replacement therapy (RRT) were still at the highest risk of death [3].

There has not been any established methodology to predict the outcomes in this high-risk population. Although several clinical scores, such as Sequential Organ Failure Assessment (SOFA) scoring, are known to be associated with ICU mortality [4], their predictive power is not sufficient in patients with severe AKI requiring CVVHDF [5]. Serum creatinine at the CVVHDF initiation, which has been the golden standard for measuring renal function, was even inversely associated with mortality [6]. Furthermore, predicting the renal recovery extent in patients with CVVHDF largely depends on each physician’s experience, without consensus. Only a few studies have been performed to explore useful indicators for predicting the optimal timing of CVVHDF discontinuation.

Urinary neutrophil gelatinase-associated lipocalin (NGAL) is one of the AKI biomarkers that reflect stress or damage on kidney epithelial cells [7, 8]. Urinary biomarkers contain their strength in that we can noninvasively and repeatedly collect urine samples at different time points during ICU stay. In the AKI progression and subsequent multiorgan dysfunction, cytokine signaling also plays an important role. High mobility group box-1 protein (HMGB1) and its downstream protein, interleukin 6 (IL-6), have shown to be key molecules in animal models of severe AKI [9]. Elevated IL-6 levels have been associated with greater inflammatory response and mortality in clinical studies [10, 11]. However, there are only sparse data on these cytokine levels in patients with severe AKI.

## Methods

### Aim

In the present study, we hypothesized that implementing urinary and plasma biomarkers during CVVHDF would contribute to predicting clinical outcomes in patients with severe AKI. This study aimed to investigate the possible roles of urinary NGAL, plasma IL-6, and HMGB1 levels in patients with severe AKI with CVVHDF.

### Study design and population

This was a multicenter prospective observational study conducted in seven university-affiliated hospitals across Japan. The patient enrollment was conducted in the following hospitals: The University of Tokyo Hospital, Tokyo Women’s Medical University Yachiyo Medical Center, Chiba University Hospital, University of Yamanashi Hospital, Teikyo University Chiba Medical Center, Wakayama Medical University Hospital, and Fujita Health University Hospital between December 2017 and September 2020.

Adult ICU patients were enrolled in the study when each on-site physician decided to start CVVHDF for AKI. The diagnosis of AKI and its treatment with CVVHDF were based on the KDIGO AKI guideline (2012) [12] and Surviving Sepsis Campaign guideline (2016) [13]. Baseline creatinine value was obtained from premorbid laboratory data acquired in previous hospital visits. For those without preceding outpatient data, the Modification of Diet in Renal Disease study formula for the Japanese population [14] was used to estimate baseline creatinine value corresponding to glomerular filtration rate of 75 ml/min/1.73 m^2^. The present study did not have any restrictions on the CVVHDF procedure including its modality, hemofilter, and intensity. Those with a modality specifically intended to reduce circulating cytokine concentrations were excluded from the study.

Patients younger than 18 years old, those on maintenance dialysis for end-stage renal disease, those receiving RRT before their ICU admission, and those with an extremely short life expectancy of less than 24 h at ICU admission were excluded. We conducted this study compiled with the tenet of the Declaration of Helsinki. Written informed consent was obtained from the participants or their next of kin. The study protocol was first approved by the Ethical Research Review Board of The University of Tokyo (registration number: 11561) and subsequently by ethical boards in all the other participating universities.

### Study protocol and definitions

**Fig. 1:**
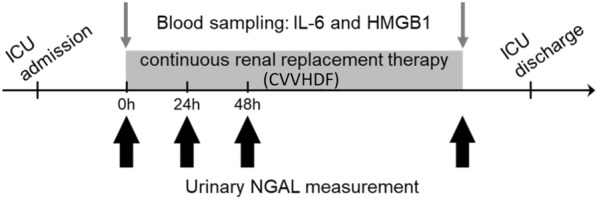
Study outline. Consecutive adult patients with severe AKI requiring CVVHDF were enrolled. Blood samples were collected at CVVHDF initiation and discontinuation. Urinary NGAL measurement was conducted at initiation, 24 h, 48 h later, and at CVVHDF discontinuation

Each on-site healthcare provider collected data on clinical characteristics (demographic data, past medical history, and clinical laboratory results), CVVHDF procedure information, and participant outcomes. Sepsis was defined according to the sepsis 3 definition [15]. We collected urine and blood samples at CVVHDF initiation and discontinuation, immediately centrifuged them to remove sediment, and stored them at − 80 ℃. Additionally, urine samples were collected at 24 and 48 h after CVVHDF initiation (Fig. 1). Urinary NGAL was subsequently measured using chemiluminescent microparticle immunoassay (ARCHITECT Urine NGAL, Abbott Japan LLC, Tokyo, Japan); plasma IL-6 and HMGB1 were measured using enzyme-linked immunosorbent assay (IL-6: TORAY Kamakura Techno‒Science Inc., Kanagawa, Japan, HMGB1: FUSO Pharmaceutical Industries Ltd., Osaka, Japan). All the assays were conducted according to the manufacturer’s instructions. Each on-site physician did not know the result of biomarker values until the end of the study.

The outcomes of this study were survival until the first CVVHDF discontinuation (CVVHDF survival) and successful CVVHDF discontinuation, which was defined as survival for 7 days without restarting CVVHDF or intermittent RRT.

### Statistical analysis

The baseline characteristics of the continuous variables were expressed as medians with interquartile ranges, and the categorical variables were expressed as counts with percentages. Wilcoxon test and Pearson’s Chi-square test were implemented in comparing differences in continuous and categorical variables, respectively. Serial change of urinary NGAL levels in each patient was tested using paired t-test. Multivariate logistic regression analysis was performed for covariate adjustment. The optimal cutoff value for predicting successful CVVHDF discontinuation was investigated by the receiver-operating characteristic (ROC) curve using Youden’s index. We used DeLong’s test and Net Reclassification Improvement (NRI)/Integrated Discrimination Improvement (IDI) indices as appropriate to perform statistical comparison of ROC curves [16]. The JMP Pro software (version 16.2.0, SAS Institute, Cary, NC, USA) was used for statistical analysis, in which p values of less than 0.05 were considered significant.

## Results

### Patients and baseline characteristics

During the observational period, 140 adult patients with AKI on CVVHDF in the seven ICUs were enrolled in the study. Seven patients with cytokine absorption modality (PMX-DHP: *n* = 4, SHEDD-fA: *n* = 3) were excluded from further analysis. Table 1 describes the baseline characteristics among the patients. Their median age was 67 years old, and they were predominantly (67.7%) men. The total ICU and in-hospital mortality in this cohort were 25.6 and 35.3%, respectively. The CVVHDF median duration was 71 h. Additional file 1: Table S1 contains a summary of detailed prescription and CVVHDF settings.

**Table 1:** Baseline characteristics

Characteristics	Overall (*n* = 133)	CVVHDF survivors (*n* = 105)	CVVHDF nonsurvivors (*n* = 28)
Age, year	67 [52, 74.5]	67 [51, 75]	68 [59, 73.5]
Men, *n* (%)	90 (67.7)	71 (67.6)	19 (67.9)
BMI, kg/m^2^	23.8 [20.3, 26.9]	24.8 [21.1, 27.2]	21.0 [19.1, 25.7]
Source of ICU admission, *n* (%)			
Emergency department	43 (32.3)	31 (29.5)	12 (42.9)
Transfer from other hospitals	19 (14.3)	17 (16.2)	2 (7.1)
Post-surgery	29 (21.8)	26 (24.8)	3 (10.7)
General ward	42 (31.6)	31 (29.5)	11 (39.3)
Comorbidity, *n* (%)			
Diabetes	41 (30.8)	33 (31.4)	8 (28.6)
Hypertension	47 (35.3)	39 (37.1)	8 (28.6)
Baseline SCr, mg/dl	0.83 [0.74, 1.09]	0.85 [0.75, 1.29]	0.79 [0.72, 0.87]
SCr at CVVHDF initiation, mg/dl	2.87 [1.66, 3.95]	3.00 [1.66, 4.16]	2.33 [1.66, 3.42]
SOFA score at ICU admission	10 [7, 13]	9 [6, 12]*	13 [10, 15]*
Patient status at CVVHDF initiation			
SOFA score	11 [8.5, 14]	11 [8, 14]*	14 [12, 17]*
Use of ventilator, *n* (%)	94 (70.7)	67 (63.8)*	27 (96.4)*
Use of vasopressor(s), *n* (%)	95 (71.4)	68 (64.8)*	27 (96.4)*
Use of diuretic(s), *n* (%)	42 (31.6)	39 (37.1)*	3 (10.7)*
Possible AKI cause, *n* (%):			
Sepsis	78 (58.6)	56 (53.3)*	22 (78.6)*
Dehydration or bleeding	43 (32.3)	32 (30.5)	11 (39.3)
Heart failure	35 (26.3)	30 (28.6)	5 (17.9)
Surgery	27 (20.3)	26 (24.8)*	1 (3.6)*
Drug	10 (7.5)	7 (6.7)	3 (10.7)
Effluent flow rate of CVVHDF /BW, ml/h/kg	22.9 [17.1, 30.7]	22.3 [16.9, 28.7]	26.8 [17.2, 40.8]
Cumulative fluid balance since ICU admission, ml	856 [54.6,3349] (range: − 5513, 21275)	639 [20.5, 3349]*	2229 [658, 4379]*
Duration of CVVHDF, hour	68 [39.5, 213]	74 [42, 206]	56 [27, 214]

Data are displayed as n (%) or median [interquartile range]

*AKI* acute kidney injury; *BMI* body mass index; *BW* body weight; CVVHDF, continuous venovenous hemodiafiltration; *ICU* intensive care unit; *NGAL* neutrophil gelatinase-associated lipocalin; *SCr* serum creatinine; *SOFA* Sequential Organ Failure Assessment

^*^p < 0.05, between survivors and nonsurvivors

### Survival status on CVVHDF discontinuation

**Fig. 2:**
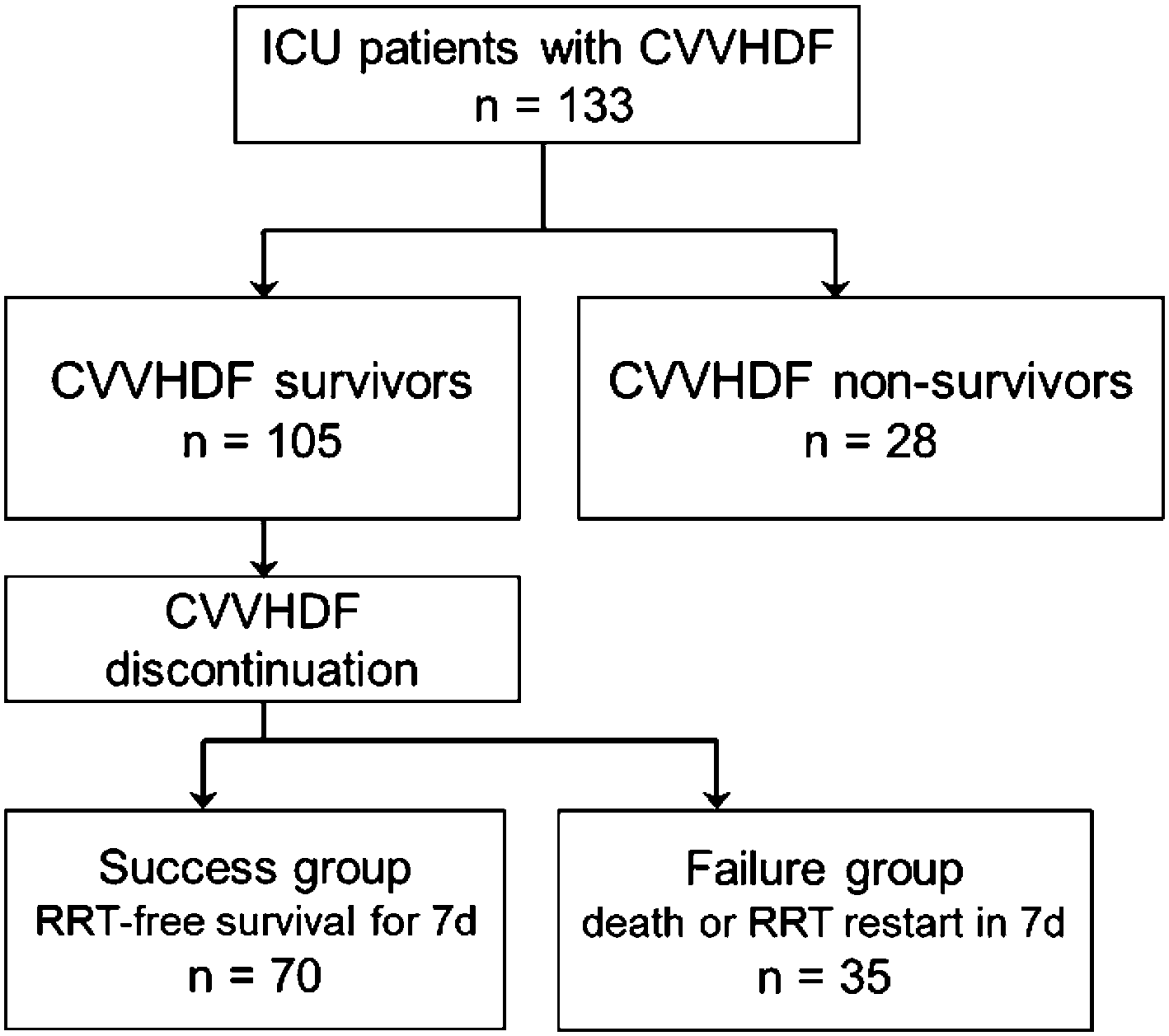
Study flow

Out of the 133 patients, 105 patients recovered from the initial AKI state, and their physician decided to stop CVVHDF (CVVHDF survivors). The others died without CVVHDF discontinuation, or their physician decided to withdraw CVVHDF for shifting to palliative care (CVVHDF nonsurvivors, Fig. 2). Table 1 shows the baseline characteristics of CVVHDF survivors and nonsurvivors. Of note, cumulative fluid balance from ICU admission until CVVHDF initiation was significantly larger in the CVVHDF nonsurvivors.

**Fig. 3:**
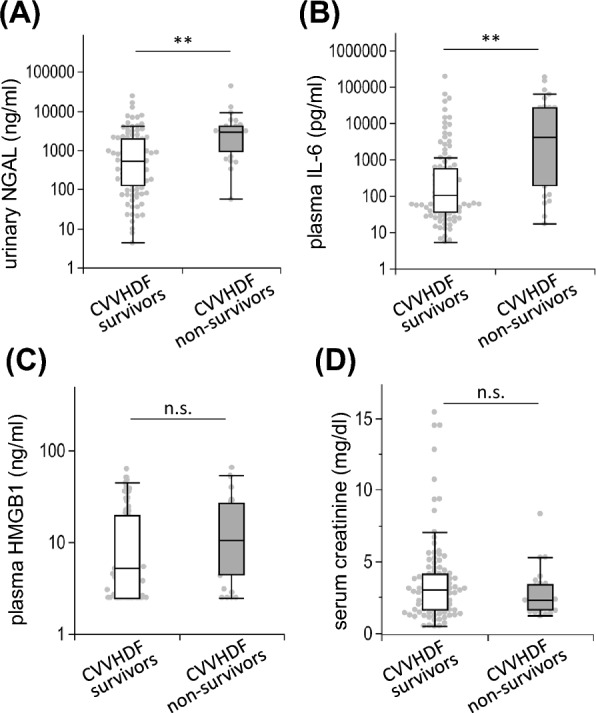
Urinary and plasma biomarkers at CVVHDF initiation. Urinary NGAL (**A**), plasma IL-6 (**B**), plasma HMGB1 (**C**), and serum creatinine (**D**) at CVVHDF initiation were compared between CVVHDF survivors and nonsurvivors during the initial CVVHDF period. Urinary NGAL and plasma IL-6 were significantly higher in CVVHDF nonsurvivors (**A**, **B**), whereas there was no significant difference in plasma HMGB1 and serum creatinine (**C**, **D**) *, *p* < 0.05; **, *p* < 0.01; n.s., not significant

Urinary NGAL and plasma IL-6 levels were significantly elevated in CVVHDF nonsurvivors (NGAL: 2941 [interquartile range (IQR): 935–4245] vs. 543 [128–1945] ng/ml, *p* < 0.01; IL-6: 4148 [192–26576] vs. 106 [36.8–567] pg/ml, *p* < 0.01, all values for CVVHDF nonsurvivors vs. survivors, Fig. 3A, B). The differences in urinary NGAL and plasma IL-6 levels by CVVHDF survival status were still significant in multivariate logistic regression analysis adjusted for age and disease severity (Additional file 1: Table S2). Neither plasma HMGB1 nor serum creatinine was associated with CVVHDF survival (HMGB1: 10.6 [IQR: 4.4–27.1] vs. 5.2 [2.5–19.8] ng/ml, *p* = 0.07; creatinine: 2.33 [1.66–3.42] vs. 3.00 [1.66–4.16] mg/dl, *p* = 0.26, all values for CVVHDF nonsurvivors vs. survivors, Fig. 3C, D). In a subgroup of patients with sepsis at CVVHDF initiation (*n* = 78), the difference in urinary NGAL and plasma IL-6 levels between the nonsurvivors and survivors remained significant (NGAL: 2997 [IQR: 1276–5158] vs. 968 [277–3311], *p* = 0.02; IL-6: 6427 [474–32875] vs. 251 [59.6–2528] pg/ml, *p* < 0.01, Additional file 1: Figure S1 A), although values were higher in the septic cohort compared to the overall cohort.

**Fig. 4:**
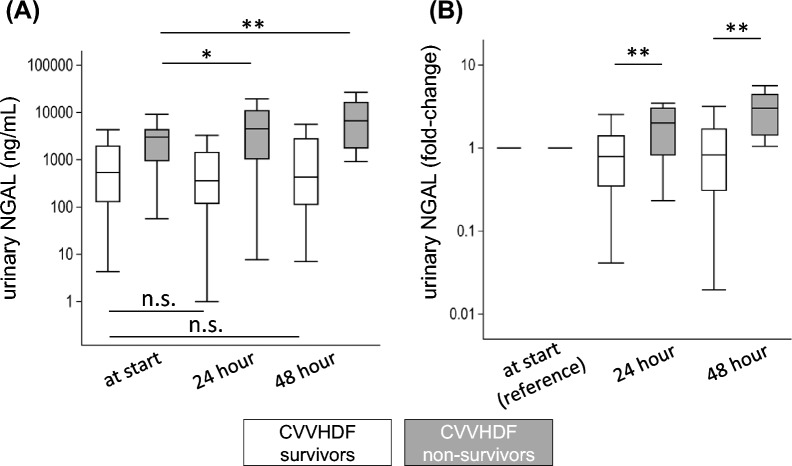
Serial measurement of urinary NGAL during CVVHDF. Urinary NGAL level was measured at initiation, 24 h, and 48 h after CVVHDF initiation. The results are shown in absolute value (**A**), and relative fold change from the initiation (**B**). **A** In CVVHDF nonsurvivors, urinary NGAL levels significantly increased at 24 and 48 h after CVVHDF initiation. Conversely, this increase was not observed in survivors. **B** The relative change of urinary NGAL from the CVVHDF initiation was significantly higher in CVVHDF nonsurvivors than in CVVHDF survivors. *, *p* < 0.05; **, *p* < 0.01; n.s., not significant

Serial measurement of urinary NGAL levels demonstrated different trends between the CVVHDF survivors and nonsurvivors. At 24 and 48 h from CVVHDF initiation, urinary NGAL levels were significantly elevated in CVVHDF nonsurvivors but not in CVVHDF survivors (Fig. 4A). The relative change of urinary NGAL from CVVHDF initiation was also significantly higher throughout the first 48 h of the CVVHDF period in CVVHDF nonsurvivors than in CVVHDF survivors (Fig. 4B).

### Success and failure in CVVHDF discontinuation

**Fig. 5:**
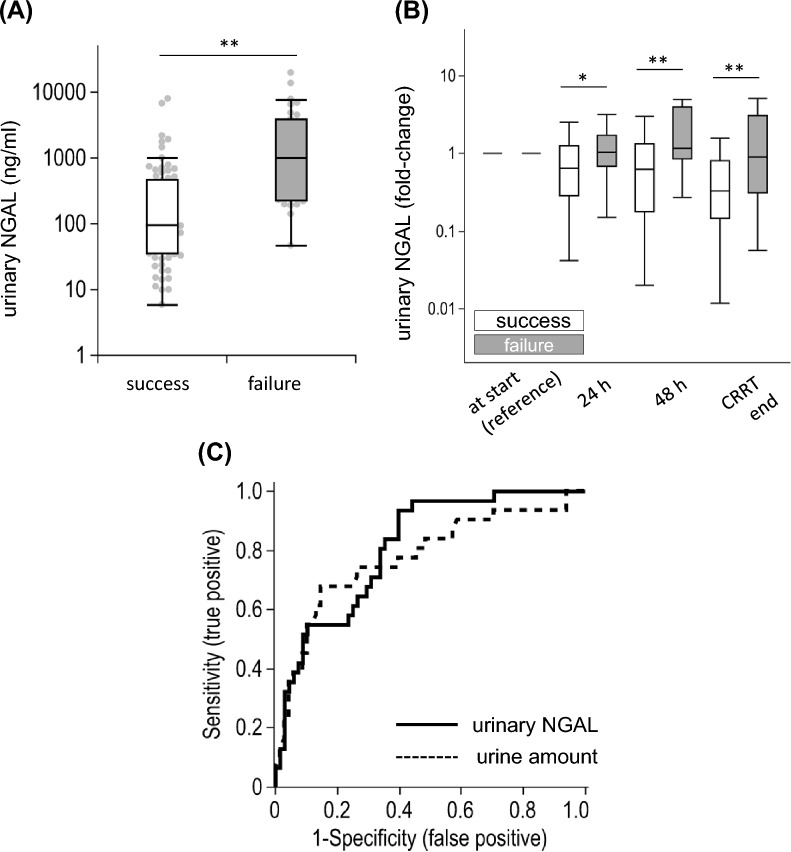
Urinary NGAL and successful CVVHDF discontinuation. **A** Urinary NGAL level at CVVHDF discontinuation was significantly lower in those who survived without restarting renal replacement therapy for 7 days from the discontinuation (success group). **B** The relative change of urinary NGAL from the CVVHDF initiation was significantly lower in the success group throughout the CVVHDF period. **C** The ROC curves were described to predict successful CVVHDF discontinuation by urinary NGAL (solid line) and urine amount (dashed line). The areas under the ROC curve were 0.81 and 0.79 for urinary NGAL and urine amount, respectively, and they did not significantly differ (*p* = 0.5). *, *p* < 0.05; **, *p* < 0.01

We focused next on those who survived the first CVVHDF period (*n* = 105) and investigated whether they succeeded in CVVHDF discontinuation. Table 2 and Additional file 1: Table S3 present the baseline characteristics of the CVVHDF survivors and CVVHDF treatment dose. Among 105 CVVHDF survivors who could be weaned from CVVHDF, 70 were free from RRT or death in the next 7 days (success group), whereas 35 died or needed RRT again (failure group). The success group required less CVVHDF duration, with a larger urine amount at discontinuation. Urinary NGAL levels in the success group were significantly lower than in the failure group (93.8 [IQR: 36.0–469] vs. 999 [227–3775] ng/ml, *p* < 0.01, Fig. 5A). The relative change of urinary NGAL from CVVHDF initiation was also significantly lower in the success group throughout the treatment period (Fig. 5B). The absolute urinary NGAL values were significantly higher in the failure group at each time point and showed a significant decrease during the CVVHDF treatment until discontinuation in the success group, but not in the failure group (Additional file 1: Table S4). Plasma IL-6 and HMGB1 at CVVHDF discontinuation did not show significant difference between those with success and failure of discontinuation (IL-6: 29.8 [IQR: 18.2–68.5] vs. 44.8 [22.0–142] pg/ml, *p* = 0.09; HMGB1: 9.3 [2.5–14.5] vs. 3.6 [2.5–14.2] ng/ml, *p* = 0.43). A subgroup analysis of the patients with sepsis showed that the difference in urinary NGAL levels at CVVHDF discontinuation between the success and failure group remained statistically significant (183 [IQR: 58.7–609] vs. 920 [210–3210], *p* < 0.01, Additional file 1: Figure S1 B). At hospital discharge, 2 patients out of 62 surviving patients (3.2%) in the success group required RRT, compared to 14 out of 24 (58.3%) in the failure group.

**Table 2:** Baseline characteristics at CVVHDF discontinuation (CVVHDF survivors, *n* = 105)

Characteristics	Success group (*n* = 70)	Failure group (*n* = 35)	*P* value
Age, year	65 [49, 74]	68 [53, 76]	0.25
Men, *n* (%)	46 (65.7)	25 (71.4)	0.35
CVVHDF duration, hour	63 [40, 148]	140 [62, 354]	< 0.01*
Effluent flow rate of CVVHDF/BW, ml/h/kg	22.4 [18.1, 28.8]	19.4 [14.2, 24.5]	0.07
Total fluid balance during CVVHDF, ml	− 1179 [− 4499, 1661]	− 1066 [− 4169, 4402]	0.72
Urine amount, ml/day	1730 [877, 2940]	360 [74, 930]	< 0.01*
PaO_2_/FIO_2_ ratio	320 [213, 435]	317 [268, 435]	0.42
SOFA score	8 [5, 10]	8 [5, 11]	0.76
Mean arterial pressure, mmHg	83 [72, 95]	82 [74, 95]	0.96
Heart rate, /minute	89 [76, 100]	83 [70, 100]	0.34
Use of ventilator, n (%)	33 (47.1)	14 (40.0)	0.49
Use of vasopressor(s), n (%)	36 (51.4)	8 (22.9)	< 0.01*
Use of diuretic(s), n (%)	40 (57.1)	12 (34.3)	0.03*
Laboratory data			
White blood cell count,/µl	10850 [8375, 16000]	10800 [6000, 16000]	0.86
Hemoglobin, g/dl	9.2 [8.3, 10.8]	8.6 [7.9, 9.5]	0.04*
Platelet count, × 10^4^/dl	9.7 [5.2, 13.9]	14.6 [6.4, 17.3]	0.03*
Blood urea nitrogen, mg/dl	21.8 [13.0, 34.5]	39.5 [25.7, 53.1]	< 0.01*
Serum creatinine, mg/dl	1.01 [0.76, 1.79]	2.11 [1.36, 3.41]	< 0.01*
Serum potassium, mEq/l	4.2 [3.8, 4.5]	4.3 [3.7, 4.6]	0.79
Total bilirubin, mg/dl	1.4 [0.8, 4.5]	2.5 [0.7, 5.2]	0.60
C-reactive protein, mg/dl	9.0 [3.3, 18.3]	7.9 [5.4, 15.3]	0.72
Arterial pH	7.43 [7.39, 7.45]	7.43 [7.40, 7.46]	0.35
Lactate, mmol/l	1.0 [0.8, 1.5]	1.3 [0.8, 1.6]	0.60

Data are displayed as *n* (%) or median [interquartile range]

*CVVHDF* continuous venovenous hemodiafiltration; *SOFA* Sequential Organ Failure Assessment

^*^p < 0.0

The optimal cutoff value of urinary NGAL to predict successful CVVHDF discontinuation was calculated to be 186 ng/ml with sensitivity and specificity of 0.94 and 0.60, respectively. The area under the ROC curve (AUC-ROC) using urinary NGAL as a predictor was 0.81 (95% confidence interval (CI) 0.71–0.88). The optimal cutoff amount of urine at CVVHDF discontinuation to predict the successful discontinuation was 772 ml per day. There was no significant difference in AUC-ROC values for urinary NGAL and urine amount (0.81 vs. 0.79, *p* = 0.5, Fig. 5C). However, the addition of urinary NGAL to urine amount yielded higher AUC-ROC (0.82, 95%CI 0.72–0.89) and significantly improved the prediction of successful CVVHDF discontinuation, which was confirmed by calculating NRI and IDI indices (NRI: 0.68 [95%CI 0.28–1.08], *p* = 0.002; IDI: 0.11 [0.04–0.18], *p* = 0.004).

## Discussion

In this prospective observational study on patients with severe AKI treated via CVVHDF in seven ICUs, we reported that elevated urinary NGAL and plasma IL-6 at CVVHDF initiation were associated with poor prognosis. Low urinary NGAL level at CVVHDF discontinuation seemed to suggest the possibility of RRT-free survival. We demonstrated that patients with an increasing trend of urinary NGAL in the first 48 h since CVVHDF initiation were likely to die during CVVHDF and, even if they survive, were likely to fail in CVVHDF discontinuation.

Accumulating reports have shown the unacceptably high mortality of patients with severe AKI. Zarbock and colleagues reported the result of the ELAIN trial for optimizing the initiation timing of RRT, in which 220 out of 231 patients (95.2%) were finally treated by RRT, and the overall 28-day mortality was 35.5% [17]. Another larger international study on the timing of RRT in ICU, STARRT-AKI, reported a similar 28-day mortality rate of 36.2% [18]. The present study also addressed this high-risk population, whose in-hospital mortality was as high as 35.3%. Our CVVHDF cohort also shared several characteristics with previous reports. First, high serum creatinine level at CVVHDF initiation was not associated with worse outcome [6]. There was no significant difference between survivors and nonsurvivors in serum creatinine level at enrollment, and all of those with very high creatinine value of > 9 mg/dl (*n* = 7) survived. This observation was at least partially attributable to fluid overload and frailty with low muscle volume in nonsurvivors. Second, a larger cumulative fluid balance at CVVHDF initiation was associated with the worse outcome as previously reported [19]. Third, small urine amount and long CVVHDF duration were again proven to be significant predictors for failure in CVVHDF discontinuation as with preceding observations [20, 21].

Contrary to the recent intense discussion on the initiation timing of CVVHDF [17, 18], only a few reports on biomarkers have focused on outcomes and recovery of kidney function after AKI. Hoste and colleagues determined a high predictive ability of urinary C–C chemokine ligand 14 for persistent AKI in an international multicenter study on 331 ICU patients with moderate to severe AKI [22]. In another study on 64 patients with severe AKI requiring RRT, urinary ezrin and moesin, which are membrane–cytoskeleton linkers in renal epithelial cells, were associated with renal recovery at 28 days after CVVHDF initiation [23]. In our study, urinary NGAL and plasma IL-6 were associated with survival outcomes, and urinary NGAL was also associated with renal recovery after CVVHDF discontinuation. According to the result that plasma IL-6 was associated with mortality but not with success and failure in CVVHDF discontinuation, IL-6 may reflect systemic damage and inflammation, not limited to the kidney. Our results also implied that the IL-6 elevation in critically ill patients requiring CVVHDF may not be induced through the HMGB1–IL-6 axis because plasma HMGB1 level was not different between survivors and nonsurvivors.

Serum and urinary NGAL, derived from renal epithelial cells and neutrophils, reportedly reflected the damage to the kidney and showed predictive value for AKI occurrence and its prognosis [24, 25]. Urinary NGAL has also demonstrated its capability of predicting the requirement of RRT in ICU [26, 27]. Recently, some researchers tried to apply NGAL to predict the successful discontinuation of CVVHDF. Chen et al. measured serum NGAL at CVVHDF discontinuation in 110 patients and confirmed that patients without RRT restart within 7 days had lower NGAL results than those who required RRT again [28]. We observed here a similar result in urinary NGAL. Stads et al. and Thomsen et al. also demonstrated the predictive value of urinary NGAL on CVVHDF discontinuation [29, 30]. The former study included 92 patients and reported that urinary NGAL was higher in the unsuccessful group. However, there was a limitation in that the measurement was conducted at 2 days after CVVHDF discontinuation. The latter study reported the urinary NGAL usefulness to differentiate those with renal recovery after the discontinuation. However, the sample size (*n* = 54) and imputation for missing values limited its generalizability. In line with these reports, our study indicated that the urinary NGAL level at CVVHDF discontinuation reflected renal recovery during CVVHDF. Notably, the predicting ability of urinary NGAL for successful CVVHDF discontinuation was only comparable with that of urine amount when used alone (Fig. 5C), but the prediction was improved when NGAL was utilized in combination with urine amount, as confirmed by NRI/IDI indices. Future study to implement urinary NGAL into the current decision-making process is warranted to establish a more stratified approach and focused management in patients with CVVHDF.

Our serial observation of urinary NGAL at the first 48 h of CVVHDF demonstrated that an increasing trend was associated with worse outcomes. A similar observation on a longer time course exists. Srisawat et al. investigated the urinary NGAL performance on days 1, 7, and 14 in ICU patients with RRT to predict their survival [31]. They found that the largest relative reduction of urinary NGAL level was associated with better survival. Given that the NGAL molecule is rarely removed via CVVHDF [32], urinary NGAL level during CVVHDF may also serve as a real-time indicator of renal damage. Thus, we can assume the patient’s dynamic state during CVVHDF. If we notice an increasing urinary NGAL trend in a patient with CVVHDF, a careful review of the current treatment strategy might be required.

There are several limitations in the present study. First, in this observational study, the decision of CVVHDF initiation, discontinuation, and restart depended on each on-site attending physician. Although we adopted the prospective multicenter design, selection bias in initiating CVVHDF at each ICU and the personal factor of each physician remain; we cannot rule out the possibility that our cohort could include patients with non-persistent AKI. Second, this study included heterogeneity in mixed ICUs with modest sample size. Future studies must validate the utility of biomarker measurement in patients with CVVHDF in a larger setting, which allows us to focus on individual etiology. Third, plasma IL-6 and HMGB1 were only investigated at the initiation and the discontinuation of CVVHDF, and the values during CVVHDF were not acquired; as compared to urinary NGAL, the serial trends of these biomarkers were not assessed. Finally, although our findings suggested survival benefits associated with low urinary NGAL and plasma IL-6 levels, these results were not appropriate for causal inference between biomarkers and outcomes.

## Conclusions

In conclusion, we demonstrated that urinary NGAL and plasma IL-6 at CVVHDF initiation were associated with mortality, and urinary NGAL at discontinuation was a significant factor in predicting successful CVVHDF discontinuation. A serial observation of urinary NGAL may also help predict the patient’s prognosis with CVVHDF and improve patient care in ICU.

## Supplementary Information

**Additional file 1: Table S1**: CVVHDF settings for the included patients. **Table S2**: Multivariate logistic regression for survival during CVVHDF. **Table S3:** CVVHDF settings for the CVVHDF survivors. **Table S4:** Urinary NGAL levels of the CVVHDF survivors. **Figure S1: **Urinary and plasma biomarkers at initiation and discontinuation of CVVHDF in patients with sepsis.

## Abbreviations

AKI: Acute kidney injury
AUC-ROC: Area under the receiver-operating characteristic curve
CVVHDF: Continuous venovenous hemodiafiltration
HMGB1: High mobility group box-1 protein
ICU: Intensive care unit
IDI: Integrated discrimination improvement
IL-6: Interleukin 6
IQR: Interquartile range
NGAL: Neutrophil gelatinase-associated lipocalin
NRI: Net reclassification improvement
ROC: Receiver-operating characteristic
RRT: Renal replacement therapy
SOFA: Sequential Organ Failure Assessment

## Declarations

### Availability of data and materials

The datasets used and/or analyzed during the current study are available from the corresponding author on reasonable request.
