# Supplementary material for: Urinary neutrophil gelatinase-associated lipocalin and plasma IL-6 in discontinuation of continuous venovenous hemodiafiltration for severe acute kidney injury: a multicenter prospective observational study
Source: Ann Intensive Care. 2023 May 15;13:42. doi: 10.1186/s13613-023-01137-6 (PMC10185728; doi:10.1186/s13613-023-01137-6)
Supplement: Supplementary file 1 — Additional file 1: Table S1: CVVHDF settings for the included patients. Table S2: Multivariate logistic regression for survival during CVVHDF. Table S3: CVVHDF settings for the CVVHDF survivors. Table S4: Urinary NGAL levels of the CVVHDF survivors. Figure S1: Urinary and plasma biomarkers at initiation and discontinuation of CVVHDF in patients with sepsis. [file 13613_2023_1137_MOESM1_ESM.docx]

**Table S1.** CVVHDF settings for the included patients

| **Settings** | **Overall**  **(N = 133)** | **CVVHDF survivors**  **(N = 105)** | **CVVHDF nonsurvivors**  **(N = 28)** | |
| --- | --- | --- | --- | --- |
| Flow at initiation  blood flow rate, ml/min  dialysate flow rate, ml/h  filtration flow rate, ml/h | 100 [100, 120]  1000 [1000, 1000]  500 [360, 500] | 100 [100, 120]  1000 [1000, 1000]  500 [400, 500] | | 100 [100, 120]  1000 [1000, 2125]  500 [300, 500] |
| Membrane, n (%)  AN69ST  polysulfone  polymethylmethacrylate  cellulose triacetate  others | 76 (57.1)  30 (22.6)  25 (18.8)  1 (0.8)  1 (0.8) | 58 (55.2)  27 (25.7)  18 (17.1)  1 (1.0)  1 (1.0) | | 18 (64.3)  3 (10.7)  7 (25.0)  0  0 |
| Anticoagulant, n (%)  nafamostat mesylate  heparin  none | 123 (92.5)  4 (3.0)  6 (4.5) | 97 (92.4)  4 (3.8)  4 (3.8) | | 26 (92.9)  0  2 (7.1) |

Data are displayed as n (%) or median [interquartile range].

CVVHDF, continuous venovenous hemodiafiltration

**Table S2.** Multivariate logistic regression for survival during CVVHDF

| **Model** | **Adjusted odds ratio (95% CI)** | **p value** |
| --- | --- | --- |
| age, *per 10 years increment*  SOFA score, *per 1 score increment*  urinary NGAL, *per 1000 ng/mL increment* | 1.08 (0.79―1.50ngal)  1.33 (1.15―1.58)  1.10 (1.02―1.24) | 0.79  <0.01*  0.04* |
| age, *per 10 years increment*  SOFA score, *per 1 score increment*  plasma IL-6, *per 1000 pg/mL increment* | 1.09 (0.79―1.52)  1.41 (1.19―1.67)  1.03 (1.01―1.05) | 0.60  <0.01*  <0.01* |

CVVHDF, continuous venovenous hemodiafiltration; IL-6, interleukin 6; NGAL, neutrophil gelatinase-associated lipocalin; SOFA, sequential organ failure assessment; 95% CI, 95% confidence interval.

*, p < 0.05

**Table S3.** CVVHDF settings for the CVVHDF survivors (n = 105)

| **Settings** | **Success group**  **(n = 70)** | **Failure group**  **(n = 35)** | **P value** |
| --- | --- | --- | --- |
| Flow at initiation  blood flow rate, ml/min  dialysate flow rate, ml/h  filtration flow rate, ml/h | 100 [100, 120]  1000 [1000, 1000]  500 [300, 500] | 100 [100, 120]  1000 [1000, 1000]  500 [500, 500] | 0.72  0.61  0.41 |
| Flow at discontinuation  blood flow rate, ml/min  dialysate flow rate, ml/h  filtration flow rate, ml/h | 100 [100, 120]  1000 [660, 1000]  500 [420, 500] | 100 [100, 100]  1000 [500, 1000]  500 [370, 500] | 0.89  0.15  0.42 |

Data are displayed as median [interquartile range].

CVVHDF, continuous venovenous hemodiafiltration

**Table S4.** Urinary NGAL levels of the CVVHDF survivors (n = 105)

| **group** | **at initiation** | **24 hours** | **48 hours** | **at discontinuation** |
| --- | --- | --- | --- | --- |
| **Success group** | 324 [78.8, 1157] | 238 [73.4, 857] | 211 [61.4, 976] | 93.8 [36.0, 469]* |
| **Failure group** | 1044 [322, 3210] | 1076 [307, 3204] | 2598 [880, 4042] | 999 [227, 3775] |

Data are displayed as median [interquartile range].

CVVHDF, continuous veno-venous hemodiafiltration; NGAL, neutrophil gelatinase-associated lipocalin

*, p < 0.05, compared to the value at initiation (paired t-test)

**Fig. S1:** **Urinary and plasma biomarkers at initiation and discontinuation of CVVHDF in patients with sepsis**

In a subgroup of patients with sepsis, levels of urinary NGAL, plasma IL-6, and HMGB1 were compared (**A**) at CVVHDF initiation between CVVHDF survivors and nonsurvivors and (**B**) at CVVHDF discontinuation between those who were successful and unsuccessful (failure) in discontinuing CVVHDF. Urinary NGAL and plasma IL-6 were significantly higher in CVVHDF nonsurvivors at initiation (**A**), and urinary NGAL was also significantly higher in the failure group at discontinuation (**B**).
